# Complementary Musical Intervention for Patients in Palliative Care in Spain: A Randomized Controlled Trial

**DOI:** 10.3390/healthcare12191938

**Published:** 2024-09-27

**Authors:** Inmaculada Valero-Cantero, María Ángeles Vázquez-Sánchez, José Luis Casals-Sánchez, Milagrosa Espinar-Toledo, Juan Corral-Pérez, Cristina Casals

**Affiliations:** 1 Puerta Blanca Clinical Management Unit, Malaga-Guadalhorce Health District, 29004 Malaga, Spain; inmaculada.valero.sspa@juntadeandalucia.es; 2 Department of Nursing, Faculty of Health Sciences, PASOS Research Group and UMA REDIAS Network of Law and Artificial Intelligence Applied to Health and Biotechnology, University of Malaga, 29071 Malaga, Spain; mavazquez@uma.es; 3 Unidad de Reumatología, Hospital Regional Universitario de Málaga, 29010 Malaga, Spain; casalssanchez@gmail.com; 4 Rincón de la Victoria Clinical Management Unit, Malaga-Guadalhorce Health District, 29730 Malaga, Spain; milagrosa.espinar.sspa@juntadeandalucia.es; 5 ExPhy Research Group, Department of Physical Education, Instituto de Investigación e Innovación Biomédica de Cádiz (INiBICA), Universidad de Cádiz, 11519 Puerto Real, Spain; juan.corral@uca.es

**Keywords:** music, home care services, palliative therapy, symptom assessment, patient satisfaction

## Abstract

Background: Patients with advanced cancer often endure a heavy burden of symptoms, both in quantity and intensity. Complementary therapies offer potential relief in this challenging scenario. Increasing the number of randomized controlled trials provides a unique opportunity to generate rigorous data, which can be used to establish causal relationships and evaluate interventions; hence, nurses can strengthen evidence-based practices, leading to better patient outcomes and quality of care. Our study aimed to evaluate the impact of a 7-day pre-recorded music intervention on cancer symptoms and satisfaction in advanced-stage cancer patients receiving palliative care at home. Methods: This multicenter, double-blind, randomized, controlled clinical trial involved 80 Spanish cancer patients receiving palliative care at home, and was conducted from July 2020 to November 2021. The intervention group (*n* = 40) received self-selected pre-recorded music for 30 min daily over 7 days. The control group (*n* = 40) received pre-recorded basic health education sessions of equal duration and frequency. Symptoms and patient satisfaction were assessed before and after the intervention using the Edmonton Symptom Assessment System and the Client Satisfaction Questionnaire, respectively. Results: Comparing the intervention with the control group, significant improvements were observed in various symptoms: total symptom burden (*p* < 0.001), pain (*p* = 0.001), fatigue (*p* = 0.007), depression (*p* = 0.001), anxiety (*p* = 0.005), drowsiness (*p* = 0.006), appetite (*p* = 0.047), well-being (*p* ≤ 0.001), and sleep (*p* < 0.001); additionally, patient satisfaction was higher in the intervention group (*p* < 0.001). Conclusions: The 7-day pre-recorded music intervention reduced both physical and psychological symptoms in advanced-stage cancer patients receiving home-based palliative care, demonstrating significant alleviation of overall symptom burden and increased satisfaction with healthcare.

## 1. Introduction

The global cancer burden is projected to reach 28.4 million cases by 2040, a 47% increase from 2020 [1]. By 2024, nearly 10 million deaths had occurred due to cancer [2], making it the second leading cause of death worldwide. Palliative care is a specialized approach aimed at improving the quality of life for patients facing serious illnesses, addressing physical, emotional, and spiritual needs [3,4,5]. Moreover, patients with advanced cancer require palliative care as early as possible to manage their symptoms effectively [3], as they often endure a significant symptom burden, both in number and severity [4]. However, it is important to note that not all early palliative care is necessarily of high quality. Quality palliative care is characterized by comprehensive care that addresses all relevant domains, such as the physical, psychological, ethical, spiritual, and social domains, for example [5]. This distinction underscores the importance of integrating evidence-based practices into palliative care plans to ensure optimal patient outcomes.

A systematic review found that the prevalence of symptoms ranged from 3.5% to 77.8%. Notably, the most common symptoms, occurring in at least 50% of patients, included fatigue, excretory symptoms, urinary incontinence, asthenia, pain, constipation, and anxiety [6]. As the disease progresses, these symptoms typically increase in frequency and worsen [7]. Therefore, the provision of appropriate palliative care not only alleviates these symptoms but also helps reduce the number of required hospital admissions, ultimately enhancing individual well-being [8]. For all these reasons, quality palliative care is essential, especially as the number of advanced cancer patients continues to rise, driven by the increasing global prevalence of the disease and the need for early palliative care intervention [9].

The patient’s situation may be enhanced through the application of complementary therapies, which are defined as “diverse medical and health care systems, practices, and products that are not generally considered part of conventional medicine” [10]. One such complementary therapy is the use of music, an effective but underexplored intervention. According to the Nursing Intervention Classification 4400, it is defined as “the use of music to help bring about a specific change in patients’ behavior, feelings, or physiology” [11]. The musical landscape offers a diverse array of elements, including various styles, instrumentation, and the presence of lyrics, whether in live or recorded formats. Additionally, factors such as session duration, volume, and the level of patient participation—ranging from active to passive music therapy—play a significant role in shaping the therapeutic experience [11,12]. In this line, music interventions can be administered using a music medicine approach, which involves offering pre-recorded music for passive listening by healthcare professionals [12]. Importantly, music elicits an emotional response that can be either positive or negative (such as fear, trembling, joy, or sadness), depending on the individual and the type of music used. These emotional responses are generated in various brain regions, including the amygdala, hippocampus, auditory cortex, and ventral and dorsal striatum [13].

However, few clinical trials have explored the use of complementary music interventions [14,15]. While two studies have reported that music interventions can relieve physical pain in palliative care patients [16,17], this effect remains unconfirmed, as another two studies found no such benefit [11,18]. Other reported impacts of music include enhanced spiritual well-being and ego integrity, reduced distress [19], decreased tiredness and fatigue [11,16,18], reduced anxiety, greater relaxation, improved well-being, and a better quality of life [11,16,20]. Quality of life is a multidimensional concept that reflects a person’s subjective evaluation of their overall health and satisfaction with life in relation to their physical condition, psychological state, level of independence, social relationships, and personal beliefs [21]. Additionally, music interventions have been associated with decreased sleepiness [11,16,18], reduced respiratory distress [18], and improved functional status [20]. A study of adult cancer patients, though not those with advanced cancer, also reported benefits from music intervention, including reduced depression, anxiety, fatigue, and pain, as well as increased hope [22].

There have been very few clinical trials examining the use of music as an adjunctive therapy for advanced cancer patients in palliative care [14,15]. The trials that do exist vary in the types of music interventions used, as well as in the frequency and duration of these interventions. More research is needed to establish the potential benefits of music therapy; as increasing the number of randomized controlled trials offers a unique opportunity to establish causal relationships and rigorously evaluate interventions. This, in turn, would enable nurses to strengthen evidence-based practices, leading to better patient outcomes and improved quality of care.

The aim of this clinical trial was to evaluate the effects of a 7-day complementary pre-recorded music intervention on cancer symptoms and patient satisfaction with healthcare among advanced-stage cancer patients receiving palliative care at home. Our primary hypothesis is that participants in the intervention group will experience an improvement in the overall symptoms of advanced cancer after the 7-day complementary music intervention compared to the control group. The secondary hypothesis is that the intervention group will report greater satisfaction with the delivered complementary intervention compared to the control group.

## 2. Materials and Methods

### 2.1. Design

This study is a randomized, double-blind, multicenter, controlled clinical trial involving patients with advanced cancer receiving outpatient palliative care (registered at ClinicalTrials.gov, NCT04052074. Registered 9 August 2019). The study was conducted in accordance with the Declaration of Helsinki, and the clinical trial protocol was reviewed and approved by the Research Ethics Committee of the Province of Malaga on 28 March 2019 (File number: AP-0157-2018). All participants were fully informed about the study, both orally and in writing, and provided signed consent prior to participation.

### 2.2. Participants

The study participants were all patients diagnosed with advanced-stage cancer, defined as cases of cancer for which a cure is not possible [23]. These patients were receiving outpatient palliative care and were recruited from six primary care clinical management units within the Malaga-Guadalhorce Health District (Malaga, Spain). They were identified from the records of patients included in the palliative care assistance process at these units. Nurse case managers involved in the study contacted potential participants directly by phone and informed them that they could choose not to participate or withdraw at any time without their care being affected. Convenience sampling based on the register of palliative care patients in their unit was used, and calls continued until the necessary sample size was achieved. Patients were recruited between July 2020 and November 2021. During the initial contact, participants were informed about the study and assessed according to the inclusion and exclusion criteria. If the criteria were met and they agreed to participate, they were recruited into the study and asked to sign the informed consent form.

The inclusion criteria were as follows: All patients had advanced cancer, were receiving outpatient palliative care, and were over 18 years old. The exclusion criteria included patients in the last days of life (those with a medically diagnosed prognosis of last days of life, characterized by evident deterioration) [24,25], patients with dementia that might impair understanding and decision-making capacity, and patients with hearing problems. Exclusion health conditions were verified using the patients’ medical records.

### 2.3. Sample Size and Randomization

The required sample size was calculated using the Edmonton Symptom Assessment System (ESAS) [26], which showed a standard deviation of 12.36. A decrease of nine points in the ESAS score (before/after intervention) was considered clinically relevant. Assuming an alpha error of 0.5 and a beta error of 0.20, we determined that two groups of 32 patients each (an intervention/control ratio of 1:1) would be necessary. To account for potential participant loss, the sample size was increased by 20%, resulting in a total of 80 patients, equally distributed between the control and intervention groups. The sample size calculation was performed using the Epidat 4.2 program [27].

After recruitment and enrollment, participants were randomly assigned to either the control or intervention group. The randomization process involved placing a slip of paper labeled “Intervention group” into 40 opaque envelopes and another labeled “Control group” into another 40 envelopes. The envelopes were then sealed, shuffled, and numbered. Each patient was assigned a number in the order of their arrival, and the corresponding envelope was opened to assign them to the indicated group. This procedure was conducted by one of the researchers. The study was designed as a double-blind randomized trial, meaning that neither the evaluators nor the patients were aware of which group they had been assigned to.

To ensure participant blinding, participants were initially informed that the study involved an intervention using headphones and that the appropriateness of the intervention would be assessed. Once randomized, they were told about the specific intervention assigned to them but were not informed whether they were in the control or intervention group. This information was provided by the researcher responsible for randomization. There was no risk of patients interacting and discussing the interventions, as there were no group meetings, and the interventions were conducted in the patients’ homes.

For evaluator blinding, all participants used either MP3 players or mobile phones, and they were instructed not to discuss anything they heard with the evaluator. Thus, the only person who knew which group the study subjects belonged to, intervention or control, was the researcher in charge of randomization.

The study assessments were conducted by the nurse case managers of the respective primary care clinical management units through home interviews, during which the study data were collected. The investigator in charge of randomization was also a nurse case manager with extensive training in palliative care.

### 2.4. Intervention

As the music intervention was complementary, all patients received the usual standard of care. In addition, the intervention group received seven half-hour sessions of pre-recorded music on consecutive days, provided via a mobile phone or MP3 player. Each patient individually chose the musical content, which contributed to their sense of well-being. This activity was facilitated by the researcher responsible for the randomization process. To select the appropriate music, participants were contacted individually after being randomized and included in the intervention group. They were asked to choose music that made them feel good, indicating the emotions it evoked and the memories it brought to mind. Based on this information, the researcher prepared a playlist designed to create positive sensations in the participants.

Patients in the control group also received usual care, along with seven half-hour audio sessions. The audios consisted of a recording of the basic health education typically provided to all patients, serving as a reinforcement of the usual education. The basic education topics covered in the recordings included information on nutrition and hydration, medication, constipation prevention, exercise and leisure, effective communication, and skin care. These audio sessions were also conducted by the researcher responsible for randomization.

Both the control and intervention groups were advised to listen to the recordings in the morning, at a time when they could take a break. No health professional was present during the listening sessions, and participants accessed the sessions independently via mobile phone or MP3 player, without further prompting.

### 2.5. Primary Outcome

The patients’ symptoms were evaluated using the ESAS [28,29], in its Spanish-language validated version, which has good reliability with a Cronbach’s alpha of 0.86 [30]. This scale scores the presence and intensity of 10 cancer symptoms over the previous week: pain, fatigue, nausea, depression, anxiety, drowsiness, dyspnea, appetite, well-being, and sleep. Each symptom is rated on a scale from 0 to 10, where 0 indicates total absence of the symptom and 10 represents its maximum possible intensity. Symptom outcomes were also assessed on two subscales: a physical subscale, which is a composite score of pain, fatigue, nausea, drowsiness, appetite, and dyspnea, yielding a score ranging from 0 to 60; and an emotional subscale, which is a composite score of depression and anxiety. The total ESAS score is calculated by summing the scores for all 10 symptoms, resulting in a range from 0 to 100.

### 2.6. Secondary Outcome

The secondary measure considered was patient satisfaction with the healthcare received, assessed using the Client Satisfaction Questionnaire (CSQ-8) [31]. In its Spanish-language validated version, the internal consistency reliability, as measured by Cronbach’s alpha, is consistently high across four ethnic groups, ranging from 0.86 to 0.91 [32]. This self-administered questionnaire evaluates eight aspects of care, with responses scored on a Likert scale from 1 to 4 points. Items 2, 4, 5, and 8 are scored by reversing the results. The overall satisfaction rating is calculated by summing the scores, including the reversed items, resulting in a total score ranging from 8 to 32, with higher scores indicating greater satisfaction [31].

### 2.7. Data Collection and Analyses

The primary results were assessed using the ESAS, which was administered before and immediately after the 7-day intervention for both study groups. The secondary outcome, satisfaction with the healthcare received, was assessed using the CSQ-8 immediately after the intervention for both groups.

Data normality was tested prior to evaluating the results. Intragroup comparisons were conducted using the Wilcoxon signed-rank test to determine if symptoms improved after the intervention. Between-group comparisons used the independent samples *t*-test or the Mann–Whitney U-test for continuous variables and the chi-square test for categorical variables.

When the experimental group showed significant improvement (measured by subtracting baseline scores from post-intervention scores) compared to the control group (*p* < 0.05), the size of the difference was explored by calculating the effect size. This was done using the formula Z/(n)½, with Z representing the value from the Mann–Whitney test and n representing the sample size for non-normal variables. For normal variables, the effect size was determined by the statistical software through Cohen’s d, with 0.5 indicating a large effect, 0.3 a medium effect, and 0.1 a small effect [33]. Data were analyzed using IBM SPSS Statistics 23.0 [34].

## 3. Results

**Figure 1:**
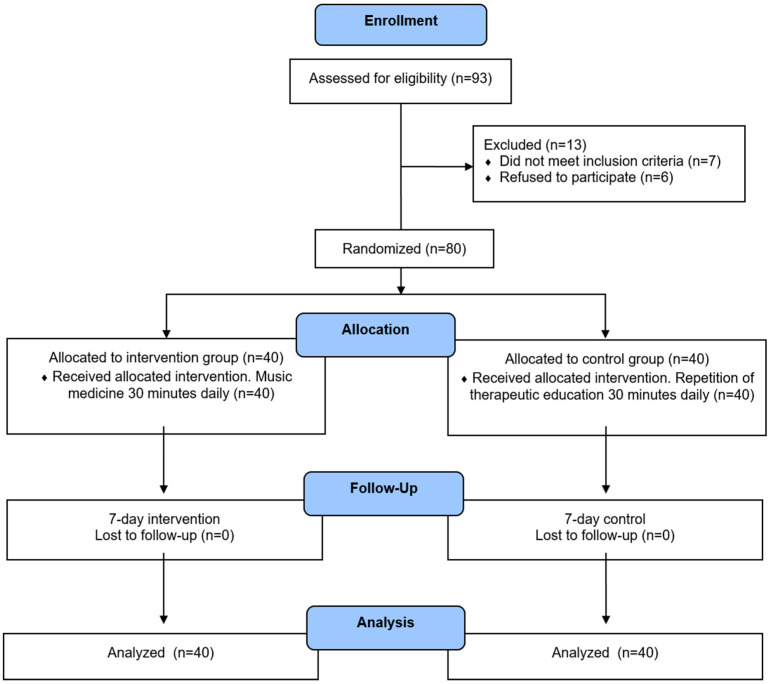
Flow chart of the study.

A total of 93 individuals were assessed for eligibility. Of these, seven did not meet the inclusion criteria, and six declined to participate in the study. Results were obtained for all 80 patients; none were lost to follow-up, and there were no adverse events. The study flowchart is presented in Figure 1.

The types of music chosen by the participants in the intervention group are described in Table 1. The most frequently selected types of music were flamenco (28%) and classical music (22%).

The sociodemographic characteristics of the participants are detailed in Table 2. The study included 55% male and 45% female participants, with a mean age of 71.83 years. The intervention group had a mean total ESAS score of 35.48 (SD = 16.58), while the control group had a mean of 38.33 (SD = 17.83). This suggests that symptom control was adequate but could be improved for patients with advanced cancer. Comparison of the initial demographic and clinical data for the 80 participants revealed no significant differences in baseline characteristics between the control and intervention groups.

Table 3 presents the before/after variation of the study variables in both the control and intervention groups. In the intervention group, several symptoms showed a trend toward improvement after the complementary therapy, including pain, nausea, depression, anxiety, drowsiness, appetite, well-being, sleep, emotional score, physical score, and total symptoms. In the control group, only nausea and dyspnea showed a trend toward improvement in the post-assessment. However, none of the changes were statistically significant.

Table 4 compares the effects of the 7-day intervention between the experimental and control groups. Consistent with our main hypothesis, the results indicate a significant difference in all variables except for nausea and dyspnea. The total ESAS score before and after the intervention for the intervention and control groups was −4.38 (SD = 9.78) and 5.68 (SD = 9.32), respectively, with a *p*-value of <0.001, indicating significant improvement in symptoms after the intervention. Regarding our secondary hypothesis, the patient satisfaction assessed using the CSQ-8 showed results of 25.90 (SD = 4.47) for the intervention group and 21.50 (SD = 4.91) for the control group, with a *p*-value of <0.001, demonstrating a high level of acceptance for the complementary therapy.

## 4. Discussion

The aim of this multicenter, double-blind, randomized, controlled clinical trial was to evaluate the potential benefits of a complementary music intervention by providing pre-recorded, self-chosen music to patients with advanced cancer receiving outpatient palliative care. The results reveal a significant improvement in the total symptom burden, and the intervention was positively received by the patients.

Among the symptoms assessed, a significant difference favoring the intervention was observed for pain compared to the control group. Previous studies on this topic have used live music in one or two 20–30 min sessions provided in a hospital setting, which differed from our approach. Two of these studies reported an improvement [16,17], while another two found no such effect [11,18]. A recent meta-analysis of advanced cancer patients suggested that music therapy can effectively improve the spiritual well-being of palliative care patients, although its beneficial effects on pain, quality of life, and psychological distress were minimal [35]. This highlights the need for more standardized research to better understand the impact of music interventions on pain in palliative care and emphasizes the effectiveness of the patient-chosen pre-recorded music intervention compared to other approaches [36]. Therefore, further research is needed with a detailed specification of the techniques used, duration, and scope to determine the most effective complementary music intervention for this patient population.

In addition, a significant difference favoring the intervention was found for other emotional symptoms, including anxiety, depression, increased well-being, and the overall emotional score, which is a composite of anxiety and depression scores. Similar findings have been observed in other studies, though these studies often used different types of complementary music therapy interventions [11,16,20,37,38]. Furthermore, the 7-day self-chosen pre-recorded music intervention significantly reduced fatigue compared to the control group. Improvements in fatigue have been reported in other research involving complementary therapies for patients with advanced cancer, though the approaches varied from ours [16,18]. For instance, one reviewed study associated fatigue reduction with music therapy that included two sessions of relaxation exercises based on live music [11]. This suggests that the type of music may influence relaxation and fatigue reduction, even though most of the music chosen in our study was not specifically relaxing. Literature reviews have shown mixed results; while listening to music can improve fatigue, a systematic review with meta-analysis did not find a significant improvement [39]. Similarly, another systematic review of music medicine interventions in non-terminal cancer patients also did not find significant improvements [22]. While music interventions can be beneficial, their effectiveness may depend on the specific characteristics of the music used. In this line, the previous literature has suggested that certain demographic factors, the role of music in the patient’s life, and the applied music therapy methods, are associated with specific needs and subjects addressed by terminally ill patients [40].

A notable finding is the significant improvement in the total ESAS symptom assessment in favor of the intervention, with only nausea and dyspnea showing no improvement. This non-pharmacological complementary intervention, easily applied at the patient’s home, highlights the potential of music-based interventions in palliative care. Music therapy offers multifaceted support—physical, psychological, emotional, expressive, existential, and social—and its effectiveness is enhanced by the holistic approach of hospice care, with patient responses highlighting specific benefits for different patient types [41]. Other symptoms that showed significant improvement favoring the intervention included appetite, sleepiness, sleep, and the physical score. Improvements in sleepiness were also noted in other studies, though different music interventions were used [11,16,18]. The observed improvements in appetite, sleep, and physical score have not been extensively described in prior studies [39]. However, sleep improvement in cancer patients has been observed and could be a beneficial aspect of the overall approach, though it has not been specifically studied in palliative care patients [42]. Given that this area is not extensively studied, further evaluation of its effectiveness is crucial.

Regarding the secondary outcome of patient satisfaction with the healthcare received, the intervention group reported significantly higher scores than the control group. The double-blind nature of the study supports that the positive reception of the complementary music intervention with pre-recorded, self-chosen music is genuine. This underscores the importance of incorporating patient preferences into therapeutic approaches to enhance overall satisfaction. Patient satisfaction is a crucial aspect of any complementary therapy, as it must be positively valued by patients to be considered effective alongside usual care.

The study results demonstrated significant differences favoring the intervention group in terms of most of the physical and psychological symptoms studied, as well as greater satisfaction with the healthcare received. These findings highlight the potential of pre-recorded complementary music interventions to enhance patient care in a home setting. For clinical practice, it is important to note that the pre-recorded complementary music intervention is easy to implement at home, has no negative consequences, and can be seamlessly integrated into nursing care. This suggests that such an intervention could be a valuable addition to standard palliative care practices.

## 5. Limitations

This clinical trial has several limitations. First, all participants were recruited from the public health system in a non-rural environment from the province of Malaga (Spain), which may limit the generalizability of the findings to other settings or populations. Additionally, the relatively small sample size (80 cancer patients) restricts the representativeness of the findings for the broader cancer patient population. Second, the study did not account for differences in cancer type, life expectancy, or medications prescribed, which could affect the outcomes. Third, the reliance on self-reported measures may introduce bias, as participants’ perceptions of symptoms and satisfaction can be influenced by various factors. Furthermore, the intervention lasted only 7 days, which may limit the sustainability of the observed effects. These limitations highlight the need for further research to explore these variables with larger sample sizes across multiple centers in Spain or internationally, thereby enhancing the understanding of palliative care interventions. Although the study was conducted after the COVID-19 pandemic, there were no substantial changes; the only minor adjustment was that nurses had to wear protective equipment, such as masks, during visits. Despite these limitations, the randomized, double-blind design strengthens the study’s internal validity.

## 6. Conclusions

The clinical trial we report highlights the benefits of providing a 7-day complementary music intervention (receptive, using pre-recorded, individually chosen music) for alleviating cancer symptoms in advanced-stage cancer patients receiving palliative care at home. The comparison between the control group and the intervention group demonstrated a significant improvement in the total ESAS symptom assessment favoring the intervention. Additionally, the self-chosen music intervention was associated with greater patient satisfaction regarding the healthcare received in this population.

## Figures and Tables

**Table 1:** Types of self-chosen music selected by palliative cancer patients.

Type of Music	N %
Flamenco singing:	14 (28%)
- “Bulerías”	3
- “Fusión flamenca”	3
- “Sevillanas”	3
- “Cante grande” or “cante jondo”	2
- “Fandangos”	2
- “Soleás”	1
Classical	11 (22%)
Latin pop	6 (12%)
Blues	4 (8%)
Pop	4 (8%)
Opera	3 (6%)
Soul	2 (4%)
Romantic ballad	2 (4%)
Disco music	2 (4%)
Rock and roll	2 (4%)

**Table 2:** Socio-demographic characteristics of patients with advanced cancer.

Characteristics		Total (*n* = 80)	Intervention Group (*n* = 40)	Control Group (*n* = 40)	*t/Z*/χ^2^	*p*
Age (years; M and SD):		71.83 (7.77)	70.51 (7.16)	73.14 (8.22)	t = −1.528	0.131
Sex (n and %):					χ^2^ = 1.818	0.178
Male		44 (55.0%)	25 (62.5%)	19 (47.5%)		
Female		36 (45.0%)	15 (37.5%)	21 (52.5%)		
Education (n and %):					χ^2^ = 0.731	0.866
No formal education		16 (20.0%)	7 (17.5%)	9 (22.5%)		
Primary		35 (43.8%)	17 (42.5%)	18 (45.0%)		
Secondary		19 (23.7%)	10 (25.0%)	9 (22.5%)		
University		10 (12.5%)	4 (10.0%)	4 (10.0%)		
Marital status (n and %):					χ^2^ = 4.292	0.117
Single		11 (13.8%)	8 (20.0%)	3 (7.5%)		
Married		51 (63.7%)	26 (65.0%)	25 (62.5%)		
Widowed/Divorced		18 (22.5%)	6 (15.0%)	12 (30.0%)		
Type of cancer (*n* and %):						
Colon		14 (17.5%)	8 (20.0%)	6 (15.0%)		
Lung		11 (13.8%)	5 (12.5%)	6 (15.0%)		
Breast		8 (10.0%)	4 (10.0%)	4 (10.0%)		
Pancreatic		7 (8.8%)	3 (7.5%)	4 (10.0%)		
Rectal		6 (7.5%)	4 (10.0%)	2 (5.0%)		
Prostate		5 (6.3%)	2 (5.0%)	3 (7.5%)		
Liver		5 (6.3%)	2 (5.0%)	3 (7.5%)		
Oropharyngeal		5 (6.3%)	3 (7.5%)	2 (5.0%)		
Kidney		4 (5.0%)	2 (5.0%)	2 (5.0%)		
Lymphoma		4 (5.0%)	3 (7.5%)	1 (2.5%)		
Bladder		3 (3.8%)	1 (2.5%)	2 (5.0%)		
Brain		3 (3.8%)	1 (2.5%)	2 (5.0%)		
Cervical		3 (3.8%)	1 (2.5%)	2 (5.0%)		
Ovarian		2 (2.5%)	1 (2.5%)	1 (2.5%)		
Months in palliative care	(Median-IQR):	3.00 (3.00)	3.00 (3.00)	2.50 (4.00)	Z = −0.982	0.326
	(M-SD):	4.59 (5.52)	5.28 (6.75)	3.90 (3.89)		
ESAS Symptom:						
Pain	(Median-IQR):	4.00 (6.00)	3.00 (5.00)	4.50 (6.00)	Z = −0.920	0.358
	(M-SD):	3.95 (2.98)	3.65 (3.03)	4.25 (2.93)		
Fatigue	(Median-IQR):	6.00 (5.00)	6.00 (4.00)	5.50 (5.00)	Z = −0.194	0.846
	(M-SD):	5.43 (2.73)	5.38 (2.61)	5.48 (2.88)		
Nausea	(Median-IQR):	0.00 (1.00)	0.00 (2.00)	0.00 (1.00)	Z = −1.114	0.265
	(M-SD):	1.18 (2.28)	1.33 (2.21)	1.03 (2.37)		
Depression	(Median-IQR):	4.00 (7.00)	3.00 (7.00)	4.00 (7.00)	Z = −1.148	0.251
	(M-SD):	4.04 (3.26)	3.63 (3.28)	4.45 (3.23)		
Anxiety	(Median-IQR):	2.00 (6.00)	1.50 (5.00)	2.50 (7.00)	Z = −1.094	0.274
	(M-SD):	3.20 (3.25)	2.93 (3.24)	3.48 (3.28)		
Drowsiness	(Median-IQR):	4.00 (6.00)	4.50 (6.00)	4.00 (5.00)	Z = −0.509	0.611
	(M-SD):	4.05 (3.08)	4.23 (3.17)	3.88 (3.01)		
Appetite	(Median-IQR):	5.00 (6.00)	4.50 (5.00)	5.00 (7.00)	Z = −0.820	0.412
	(M-SD):	4.29 (3.13)	4.00 (2.89)	4.58 (3.36)		
Well-being	(Median-IQR):	5.00 (4.00)	5.00 (4.00)	5.00 (3.00)	Z = −0.441	0.659
	(M-SD):	5.43 (2.42)	5.28 (2.56)	5.58 (2.29)		
Dyspnea	(Median-IQR):	0.00 (4.00)	1.00 (2.00)	0.00 (4.00)	Z = −0.307	0.759
	(M-SD):	2.00 (2.88)	1.88 (2.66)	2.13 (3.12)		
Sleep	(Median-IQR):	3.00 (6.00)	2.00 (6.00)	3.00 (4.00)	Z = −0.702	0.483
	(M-SD):	3.35 (2.85)	3.20 (3.13)	3.50 (2.56)		
Emotional Score	(Median-IQR):	6.00 (10.00)	5.00 (12.00)	7.00 (11.00)	Z = −1.119	0.263
	(M-SD):	7.24 (6.05)	6.55 (6.07)	7.93 (6.03)		
Physical score (M-SD):		20.89 (10.38)	20.45 (9.04)	21.33 (11.67)	t = −0.375	0.709
Total symptoms (M-SD):		36.90 (17.17)	35.48 (16.58)	38.33 (17.83)	t = −0.740	0.461

Abbreviations: ESAS, Edmonton Symptom Assessment System; IQR, interquartile range; M, mean; SD, standard deviation. Significance was set at *p* < 0.05.

**Table 3:** Variation of before–after study variables in the intervention and control groups.

Variables	Experimental Group (*n* = 40)				Control Group (*n* = 40)			
	Pre	Post	*t/Z*	*p*	Pre	Post	*t/Z*	*p*
Total symptoms:								
(M-SD):	35.48 (16.58)	31.10 (15.14)	t = −1.233	0.221	38.33 (17.83)	44.00 (18.05)	t = 1.415	0.161
ESAS Symptom:								
Pain								
(Median-IQR):	3.00 (5.00)	2.00 (6.00)	Z = −0.485	0.627	4.50 (6.00)	5.00 (5.00)	Z = −1.635	0.102
(M-SD):	3.65 (3.03)	3.38 (3.09)			4.25 (2.93)	5.38 (2.83)		
Fatigue								
(Median-IQR):	6.00 (4.00)	6.00 (4.00)	Z = −0.019	0.985	5.50 (5.00)	6.50 (5.00)	Z = −1.173	0.241
(M-SD):	5.38 (2.61)	5.40 (2.64)			5.48 (2.88)	6.25 (2.72)		
Nausea								
(Median-IQR):	0.00 (2.00)	0.00 (2.00)	Z = −0.920	0.358	0.00 (1.00)	0.00 (1.00)	Z = −0.230	0.818
(M-SD):	1.33 (2.21)	0.93 (1.76)			1.03 (2.37)	0.95 (2.09)		
Depression								
(Median-IQR):	3.00 (7.00)	1.50 (5.00)	Z = −1.513	0.130	4.00 (7.00)	5.00 (5.00)	Z = −1.411	0.158
(M-SD):	3.63 (3.28)	2.55 (2.68)			4.45 (3.23)	5.40 (3.08)		
Anxiety								
(Median-IQR):	1.50 (5.00)	3.00 (5.00)	Z = −0.040	0.968	2.50 (7.00)	3.00 (8.00)	Z = −1.012	0.311
(M-SD):	2.93 (3.24)	2.73 (2.67)			3.48 (3.28)	4.18 (3.56)		
Drowsiness								
(Median-IQR):	4.50 (6.00)	4.50 (5.00)	Z = −0.656	0.512	4.00 (5.00)	4.00 (6.00)	Z = −0.417	0.677
(M-SD):	4.23 (3.17)	3.80 (2.95)			3.88 (3.01)	4.20 (3.13)		
Appetite								
(Median-IQR):	4.50 (5.00)	3.00 (6.00)	Z = −0.851	0.395	5.00 (7.00)	5.00 (7.00)	Z = −0.310	0.757
(M-SD):	4.00 (2.89)	3.50 (3.23)			4.58 (3.36)	4.83 (3.43)		
Well-being								
(Median-IQR):	5.00 (4.00)	5.00 (4.00)	Z = −1.136	0.256	5.00 (3.00)	6.00 (4.00)	Z = −1.432	0.152
(M-SD):	5.28 (2.56)	4.73 (2.39)			5.58 (2.29)	6.40 (2.46)		
Dyspnea								
(Median-IQR):	1.00 (2.00)	0.00 (3.00)	Z = −0.480	0.632	0.00 (4.00)	0.00 (4.00)	Z = −0.124	0.901
(M-SD):	1.88 (2.66)	1.90 (2.90)			2.13 (3.12)	2.10 (3.28)		
Sleep								
(Median-IQR):	2.00 (6.00)	1.50 (4.00)	Z = −1.397	0.162	3.00 (4.00)	4.00 (5.00)	Z = −1.216	0.224
(M-SD):	3.20 (3.13)	2.20 (2.40)			3.50 (2.56)	4.33 (3.01)		
Emotional score:								
(Median-IQR):	5.00 (12.00)	5.00 (9.50)	Z = −0.705	0.481	7.00 (11.00)	8.00 (9.75)	Z = −1.265	0.206
(M-SD):	6.55 (6.07)	5.28 (4.83)			7.93 (6.03)	9.58 (6.17)		
Physical score:								
(M-SD):	20.45 (9.04)	18.90 (9.44)	t = −0.750	0.456	21.33 (11.67)	23.70 (11.35)	t = 0.923	0.359

Abbreviations: ESAS, Edmonton Symptom Assessment System; IQR, interquartile range; M, mean; SD, standard deviation. Significance was set at *p* < 0.05.

**Table 4:** Comparison between intervention and control group in relation to study variables.

Outcome		Intervention Group (*n* = 40)	Control Group (*n* = 40)	*t/Z*	*p*	Effect Size
Total symptoms (M-SD):		−4.38 (9.78)	5.68 (9.32)	t = −4.705	<0.001	0.93
ESAS Symptom:						
Pain	(Median-IQR):	0.00 (2.00)	1.00 (2.00)	Z = −3.183	0.001	0.50
	(M-SD):	−0.28 (1.91)	1.13 (1.86)			
Fatigue	(Median-IQR):	0.00 (2.00)	1.00 (1.00)	Z = −2.719	0.007	0.43
	(M-SD):	0.03 (1.95)	0.78 (1.40)			
Nausea	(Median-IQR):	0.00 (0.00)	0.00 (0.00)	Z = −1.524	0.127	0.24
	(M-SD):	−0.40 (1.15)	−0.08 (1.02)			
Depression	(Median-IQR):	0.00 (1.00)	1.00 (1.75)	Z = −4.454	<0.001	0.70
	(M-SD):	−1.08 (2.60)	0.95 (1.45)			
Anxiety	(Median-IQR):	0.00 (1.00)	0.00 (1.00)	Z = −2.840	0.005	0.45
	(M-SD):	−0.20 (2.70)	0.70 (1.77)			
Drowsiness	(Median-IQR):	0.00 (2.00)	0.00 (1.00)	Z = −2.774	0.006	0.44
	(M-SD):	−0.43 (1.93)	0.33 (1.58)			
Appetite	(Median-IQR):	0.00 (2.75)	0.00 (1.00)	Z = −1.987	0.047	0.31
	(M-SD):	−0.50 (2.38)	0.25 (2.06)			
Well-being	(Median-IQR):	0.00 (1.00)	1.00 (2.00)	Z = −3.740	<0.001	0.59
	(M-SD):	−0.55 (1.88)	0.83 (1.80)			
Dyspnea	(Median-IQR):	0.00 (0.00)	0.00 (0.00)	Z = −0.785	0.433	0.12
	(M-SD):	0.03 (1.42)	−0.03 (0.86)			
Sleep	(Median-IQR):	0.00 (2.00)	1.00 (1.00)	Z = −4.402	<0.001	0.70
	(M-SD):	−1.00 (2.39)	0.83 (2.15)			
Emotional score:	(Median-IQR):	0.00 (2.00)	1.50 (3.00)	Z = −4.093	<0.001	0.65
	(M-SD):	−1.28 (4.39)	1.65 (2.82)			
Physical score (M-SD):		−1.55 (5.65)	2.38 (4.38)	t = −3.472	0.001	0.72
CSQ−8 (M-SD):		25.90 (4.47)	21.50 (4.91)	t = 4.193	<0.001	0.85

Abbreviations: CSQ-8, Client Satisfaction Questionnaire ESAS; Edmonton Symptom Assessment System; IQR, interquartile range; M, mean; SD, standard deviation. Significance was set at *p* < 0.05. An effect size over 0.5 indicated a large effect, 0.3 indicated a medium effect, and 0.1 indicated a small effect.

## Data Availability

Data are available upon reasonable request to the corresponding author.
